# History of Dentists Who Have Graduated under Drs. Harwood and Tucker

**Published:** 1892-05

**Authors:** E. G. Tucker


### HISTORY OF DENTISTS WHO HAVE GRADUATED UNDER DRS. HARWOOD AND TUCKER.[1]

BY DR. E. G. TUCKER.

Mr. President and Gentlemen,—It is sometimes pleasant to refer back to former times and persons. Perhaps it would be interesting to our young friends to learn what has transpired and originated from two individual dentists who were associated for many years as early as 1830,—Daniel Harwood, M.D., and Joshua Tucker,

---

[1] Read before the American Academy of Dental Science, January 9, 1892.

M.D. They were truly lovers of their profession, and desired to develop its progressiveness in all its liberal branches.

The following persons received from one or both of said doctors, in person, the principles of filling teeth with soft gold-foil and strictly hand-pressure. Each of the following dentists has left an honorable record in his different location: Dr. W. W. Couma, Boston; Dr. Blood, Worcester; Dr. S. B. Straw, Bangor; Dr. Hawes, Providence, R. I.; Dr. E. G. Tucker, Boston; Dr. Jos. H. Foster, New York; Dr. Eldridge Bacon, Portland; Dr. Horace Kimball, New York. Under the care of Joshua Tucker and E. G. Tucker were: Dr. Benj. Codman, Boston; Dr. Henry Jordon, Boston; Dr. Edward Gage, Paris; Dr. R. H. Gage, Mobile; and later, under the care of Dr. Joshua Tucker: Dr. Elisha T. Wilson, Taunton; Dr. Geo. T. Moffat, Boston; Dr. L. D. Shepard, Salem; Dr. E. P. Bradford, Boston.

I have simply enumerated facts without attempting to enlarge on individual attainments, which would take up too much of your time and are too well known to the profession to need any word of mine.

I would further state, that in 1830, the late Joshua Tucker brought from Cuba a receipt for mineral teeth, and with joint hands of the doctors and the perseverance and industry of the late W. W. Codman, M.D., in the laboratory, mineral teeth became a factor in mechanical dentistry.

